# Unrecognized schizophrenia spectrum and other mental disorders in youth disconnected from education and work-life

**DOI:** 10.3389/fpsyt.2022.1015616

**Published:** 2022-10-26

**Authors:** Line Lindhardt, Lars Siersbæk Nilsson, Povl Munk-Jørgensen, Ole Steen Mortensen, Erik Simonsen, Julie Nordgaard

**Affiliations:** ^1^Early Psychosis Intervention Center, Mental Health Services East, Psychiatry Region Zealand, Roskilde, Denmark; ^2^Mental Health Center Amager, Mental Health Services Capital Region of Denmark, Copenhagen, Denmark; ^3^Psychiatric Research Academy, Region of Southern Denmark, Odense, Denmark; ^4^Department of Occupational and Social Medicine, Holbæk Hospital, Holbæk, Denmark; ^5^Department of Public Health, Faculty of Health and Medical Sciences, University of Copenhagen, Copenhagen, Denmark; ^6^Mental Health Services East, Copenhagen University Hospital, Psychiatry Region Zealand, Roskilde, Denmark; ^7^Department of Clinical Medicine, Faculty of Health and Medical Sciences, University of Copenhagen, Copenhagen, Denmark

**Keywords:** schizophrenia spectrum disorder, NEET, psychopathology, social disconnection, psychosis

## Abstract

**Background:**

Schizophrenia spectrum disorders typically emerge during adolescence or early adulthood. Often the symptomatology is vague initially, while a marked functional decline and social withdrawal can be seen. A group of young people with such social and functional impairments is the so-called “Not in Education, Employment or Training” (NEET), i.e., a youth population that is socially disconnected from education and work-life. Despite the NEET group’s disconnection from important parts of social life and a rising concern of an intersection with mental health problems, a psychopathological perspective on the problems experienced by this group remains underexplored.

**Aim:**

To examine a NEET sample for psychopathology and if relevant allocate psychiatric diagnoses.

**Methods:**

We performed an interview study comprising 40 participants from youth job-counseling services. All underwent a comprehensive psychiatric evaluation. Inclusion criteria were 18–29 years of age and a welfare benefit history of minimum 6 months.

**Results:**

Diagnostic criteria of any mental disorder were fulfilled by 95% of the sample; half of whom were diagnosed with a schizophrenia spectrum disorder. The participants with schizophrenia spectrum disorders had lower global functioning, were more often in contact with the mental health services and had higher PANSS and Examination of Anomalous Self-Experiences (EASE) scores compared to those with non-schizophrenia spectrum disorders. The participants fulfilling the criteria for schizophrenia spectrum disorders had lower EASE and PANSS scores than usually reported in the literature, suggesting more “symptom-poor” presentations.

**Conclusion:**

Psychiatric illness and particularly schizophrenia spectrum disorders affecting social interaction and the ability to take part in educational and work-life were grossly overrepresented in the NEET sample. Our findings suggest that pronounced social disconnection in youth in and of itself should lead to suspect the presence of a severe mental disorder.

## Introduction

Social disconnection in the general youth population results in marginalization for the individual, and has considerable economic consequences for society (1). Consequently, socially marginalized youth is a major public health concern with social policy interest (2, 3). The term “Not in Employment, Education or Training” (NEET) is an international consolidated indicator and among others used by Eurostat and the Organization for Economic Co-operation and Development to describe school-to-work transition difficulties in a vulnerable population of youth facing “social exclusion” (4–6). The concept of NEET has been adopted by researchers and governmental officials to define disconnection from education and work-life in the youth population (3, 7).

Obviously, there are societal differences such as dissimilar opportunities in education and work-life and social inequality which must be taken into account when looking at NEET from a global perspective (8, 9). Moreover, particular culture-bound forms of severe social withdrawal with underexplored relations to more well-defined mental disorders have been reported, cf. the Japanese phenomenon of hikikomori (10, 11).

Studies suggest, however, that mental health problems may be common denominators of the NEET population irrespectively of country and cultural setting and after controlling for possible confounding factors such as social disadvantage (7, 12–14). Unfortunately, not much is known about mental health problems in young, non-help-seeking NEET individuals.

It is primarily large surveys and health outcome data in population-based studies that have addressed the issue (13, 15–18). They have demonstrated significant associations with depressive symptoms, anxiety symptoms and substance use (13, 15, 18) and, that NEET status in young adults is associated with mental disorders in childhood and adolescence (7, 14, 19). A recent systematic review and meta-analysis by Gariépy et al. showed that the NEET status population had an odds ratio (OR) of 1.43 for having mood disorders, an OR of 1.55 for anxiety disorders and an OR of 1.72 for any mental disorder. However, the study pointed to the need for more knowledge about mental health and NEET status. Especially pertinent is the absence of studies addressing psychotic disorders in the NEET status population (20, 21). On the other hand it is well established that young adults experiencing a first-episode psychosis, are often disconnected from education and work when entering into contact with the mental health services (22–24).

Still, social disability tends to be overlooked or perhaps even outright neglected as a characteristic and important sign for schizophrenia spectrum disorders (25). This is especially relevant in symptom-poor schizophrenia in which a prominent decrease in social functioning is often among the first signs of evolving illness (26). In a 20-year longitudinal study comprising information from 485 patients Velthorst et al. found severe and persistent social impairment to be common in the schizophrenia spectrum disorder course (75%) (27).

Critics have pointed to initiatives in early detection of psychosis not taking the significance of social impairment or functional decline fully into account and as such young people with the NEET status are suggested to be a candidate group for early intervention and preventative strategies (28, 29). By thoroughly exploring psychopathology in a NEET population our understanding of the barriers for engaging in social and work life in such groups could be widened.

## Aims

To investigate psychopathology outside the boundaries of the mental health system within a group of NEETs, and allocate a diagnosis if relevant. Additionally, to further examine the clinical characteristics of the NEETs.

## Materials and methods

### Study population

The NEET population was recruited from municipality job-counseling centers within local government social services. In Denmark, young people not in work or education are entitled to financial support if they cannot support themselves. The organization of the assessment of rights to claim financial assistance, is governed by the municipalities and organized in job-counseling centers within the social services (30). Usually, a prerequisite for receiving such financial support is partaking in meetings in the job-counseling centers and non-participation customarily results in the loss of this entitlement (31). Thus, the vast majority of NEETs are in contact with the job-counseling centers.

The participants were recruited from three municipality job-counseling centers of the region Zealand of Denmark: Roskilde Municipality (89,000 inhabitants), Køge Municipality (60,000 inhabitants) and Stevns Municipality (22,000 inhabitants). The catchment areas of these municipalities are primarily rural or suburban.

The following inclusion criteria were applied:

1. Aged 18–29 years
2. Had received non-medical welfare benefits for at least 26 consecutive weeks before the invitation to the study
3. Fluently Danish-speaking

The age range corresponded to the organization in the Danish job-counseling centers, with some organizational differences between municipalities, with specific teams handling the 18–29 year-old young adults. In this study, NEET was defined as a duration of more than 26 consecutive weeks receiving educational benefits, thereby ruling out the possibility of a more transient state of disconnection. Eligibility for participation was independent of prior or current psychiatric treatment.

### Recruitment procedure

**FIGURE 1:**
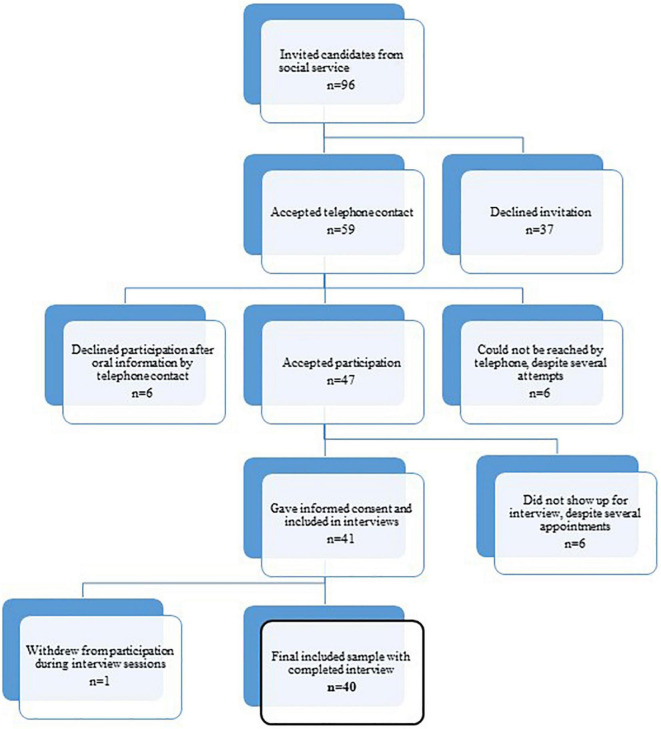
Flow of study participants.

The participants were recruited by case workers in the three municipalities, who were asked to invite all young people in the job-counseling centers fulfilling the inclusion criteria. Collaboration with the caseworkers in the social services was established by in-person meetings with the teams of caseworkers handling the youth population seeking social assistance. If the invited candidates accepted, the first author LL contacted them by phone. The interviews were conducted either in a municipality facility or in the local outpatient mental health clinics, depending on the participant’s preference. All participated upon informed consent. The recruitment and inclusion of participants are presented in Figure 1.

### Assessment

The psychopathological and diagnostic interview included:

- Operational Criteria Checklist for Psychotic Illness and Affective Illness scale (OPCRIT) (32) [an extract of the Present State Examination (PSE)].
- Positive and Negative Syndrome Scale (PANSS) (33).
- Bonn Scale for the Assessment of Basic Symptoms (BSABS), the perceptual section (34).
- Assessment of the First Rank Symptom continua (35).
- Examination of Anomalous Self-Experiences (EASE) (36).
- Global Assessment of Functioning scale, the split version (GAF-S and GAF-F) (37, 38).

The composite of scales derived from former studies exploring schizophrenia spectrum psychopathology and differential diagnosis (39–43). The participants were also assessed with respect to life history, overall psychosocial functioning, educational and vocational history including periods of welfare benefit dependence, family history of mental disorders, and the history of contact with mental health professionals, mental illness evolution and history of somatic diseases and physical symptoms with consequences for overall functioning.

EASE, a symptom checklist consisting of 57 main items, was used to explore anomalous self-experiences (36). In accordance with previous publications, the EASE items were scored only present or absent, not including severity or frequency, and subtypes were collapsed into main items (41, 44).

The timeframe for the assessment of all the scales, other than PANSS and GAF, was lifetime. The timeframe of the PANSS was, as specified by its authors, “the previous week” but did not include descriptions by peers and surrounding professionals (33). The GAF was evaluated as current state at the time of evaluation (45). The interviews were split into several sessions if needed, depending on the participant’s capability. In such cases, the interview sessions were conducted with a short interval and within a maximum of a few weeks. The interviews were conducted between 1st April 2019 and 1st January 2021.

All interviews were either videotaped or audiotaped. The interviews were semi-structured, conversational, and phenomenologically orientated, encouraging faithful self-descriptions (39, 40). Items were only rated as present if the participants provided descriptions and examples that fitted a relevant item definition, i.e., an item was not rated as present based on a simple “yes” answer. All interviews were completed and there were no missing data.

The interviews were performed by the LL, an MD with several years of experience in clinical psychiatry, certified in the application of the EASE, and tested for interrater reliability for the EASE prior to the study with JN, a senior psychiatrist and researcher and certified EASE instructor (interrater reliability κ = 0.75). Further the interviewer LL is a PSE instructor at regular courses. Average duration of interviews was 4 h [range: 2 h and 45 min–7 h and 0 min].

Finally, all patients were allocated a “Best-Estimate Life-Time” ICD-10 diagnosis by LL. If any uncertainty about psychopathological phenomena or diagnosis occurred, JN or LSN, an experienced MD and certified EASE instructor, were consulted and extracts of interview recordings were evaluated jointly to reach consensus. Additionally, randomly selected videotaped interviews were jointly evaluated with respect to general psychopathology, EASE items, and overall consensus of best-estimate life-time diagnoses. In total, consensus ratings were performed in 26 (65%) of the included interviews. All items were rated conservatively i.e., if the presence of an item was considered doubtful, this item was consistently rated as absent.

If the participants reported any lifetime substance use, the diagnosis of schizophrenia, non-affective psychosis or schizotypal disorder was only made if the psychopathology could convincingly be related to periods in which the individuals had not used any substances for more than 6 months of abstinence or significant psychopathology present prior to onset of substance use.

### Diagnostic grouping

We grouped the diagnoses according to the ICD-10 hierarchy with (1) schizophrenia and non-affective psychosis; (2) schizotypal disorder; (3) other mental disorders; (4) no mental disorder.

### Ethics

The study was evaluated by the National Committee on Health Research Ethics, journal no. 18-000080, and was concluded not to fall under the Health Research Act. The study was approved by the Danish Data Protection Agency, journal no. REG-064-2018.

If mental disorders requiring psychiatric treatment were identified during the interviews, the participants were referred to a relevant mental health service.

### Statistical analyses

We dichotomized the diagnostic variables by merging the data into diagnostic groups of schizophrenia spectrum disorders, i.e., schizophrenia, non-affective psychosis and schizotypal disorder (43), and a group of other mental disorders. We tested for association between schizophrenia spectrum disorder and social functioning as measured by GAF-F, GAF-S, and total PANSS sum score applying *t*-test. The assumption of normal distribution was tested using Shapiro-Wilk test. The GAF-S and GAF-F scales were scored as continuous scales (37). For the variables GAF-F score and PANSS sum score the assumption of normal distribution was not fulfilled, and we therefore used the non-parametric Wilcoxon Signed Rank test to test for differences between groups.

We used ANOVA to compare EASE mean scores between the diagnostic groups (schizophrenia, schizotypal disorder, other mental disorder, and no mental disorder). We corrected for multiple testing by Scheffe’s test (46).

All analyses were conducted using SAS Enterprise Guide 7.1. Significance level was set to 0.05.

## Results

A total of 40 participants were included in the study with a mean age of 24 years, 53% male.

The typical participant had received non-medical welfare benefits for more than 1 year (mean 90 weeks, range [27.8–317.3]), had in at least one prior period received non-medical welfare benefits (*n* = 26, range [1–7 episodes]), and had an educational level of compulsory education or less (48%). In the majority of cases, there was a history of frequent disruptions to educational plans or vocational attachments (73%). In addition, the typical participant lived alone and was not in a relationship (55%), had parents that suffered from mental disorders or substance use problems (71%), and the participant had been in contact with mental health services in childhood or adolescence (42%). About one third had not completed final exams in compulsory school (30%). The sample had mean GAF-F 49.0 (IQR: 45.0–58.0).

**FIGURE 2:**
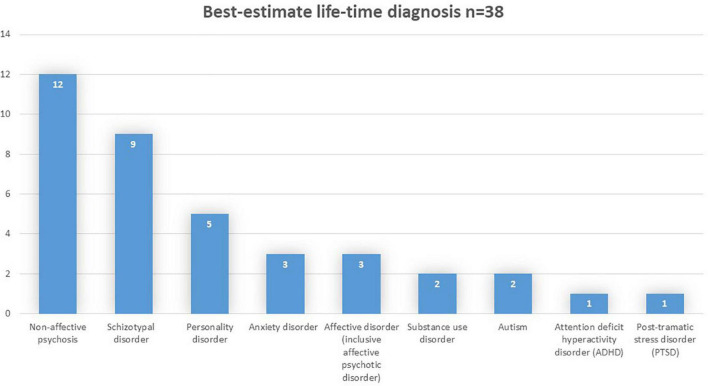
Distribution of mental disorders.

We found that 38 (95%) participants fulfilled the criteria for a lifetime diagnosis of a mental disorder, and 21 (53%) were allocated a diagnosis of schizophrenia spectrum disorder. More specifically 10 participants fulfilled the diagnostic criteria for schizophrenia, 9 for schizotypal disorder, and 2 for unspecified non-affective psychosis. The distribution of disorders among the sample is presented in Figure 2.

Only 2 participants did not meet the diagnostic criteria of any mental disorder. One of these was newly diagnosed with a chronic somatic disease that explained the reduced ability to engage in education and work. The other was during his school years suspected of having an attention deficit disorder, but the diagnostic outcome of the prior mental examination remained unclear. The interview revealed only vague symptomatology with social withdrawal as the most prominent finding, and no diagnosis of any mental disorder could be allocated in the present study.

For two participants, clear psychopathological phenomena were only present during periods with ongoing substance use, and they were therefore only assigned a diagnosis of substance use.

### The schizophrenia spectrum disorder group

Among the 21 participants who were diagnosed with a schizophrenia spectrum disorder in the study 14 were already in contact with the psychiatric services: 5 of those were diagnosed with schizotypal disorder, 2 with schizophrenia and 7 with a non-schizophrenia spectrum disorder.

The participants who fulfilled the criteria for a schizophrenia spectrum disorder had mean EASE score of 11.8 (*SD*: 1.7). The EASE mean scores were significantly higher for participants with a diagnosis of schizophrenia, other non-affective psychoses or schizotypal disorder than for participants with other mental disorders and no mental disorders (Table 1). Correspondingly, participants with schizophrenia spectrum disorders scored lower than participants with other mental disorders on GAF-F [median: 48.0 (IQR 41.0–50.0) vs. 55.0 (IQR 49.0–69.0)] and GAF-S [mean 52.1 (SD 11.3) vs. 64.8 (SD 8.0)] and higher on PANSS [median 50.0 (IQR 40.0–57.0) vs. 34.0 (IQR 31.0–41.0)]. The median PANSS positive score was 11.0 (IQR 7.0), the median PANSS negative score was 11.0 (IQR 7.0) and the median PANSS general score was 25.0 (IQR 9.0) in the subgroup.

**TABLE 1:** Distribution among the study population of diagnoses allocated by assessment, previous lifetime clinical diagnoses, and EASE scores.

	ICD-10 diagnoses			
	Schizophrenia and non-affective psychosis	Schizotypal disorder	Other non-schizophrenia spectrum disorders^§^	No diagnosis of any mental disorder
Best estimate lifetime disorders by assessment (n)	12	9	17	2
Diagnoses clinically assigned previously, lifetime (n)	2	5	24	9
EASE^α^ mean score (SD)	13.4 (6.0), [4–24]*¤	12.8 (5.2), [4–13]*¤	4.4 (3.5), [1–12]	0.5 (0.7), [1]

^§^Mental disorders not belonging the schizophrenia spectrum (schizophrenia, schizotypal disorder) or non-affective psychosis and includes substance use disorder. *Difference in mean compared to the group assessed not to fulfill criteria of any mental disorder lifetime (*p* < 0.05). ¤ Difference in mean compared to the group assessed to fulfill criteria of any lifetime non-schizophrenia spectrum disorders inclusive substance use disorder (*p* < 0.05). ^α^Examination of anomalous self-experiences.

### Clinical case illustrations

We here present two illustrative cases from the schizophrenia spectrum disorder group.

#### Case 1

A 28-year-old female grew up in a family of four. She had social difficulties throughout childhood and adolescence; she had no friends and became anxious around other people. She had a hard time keeping up with the schoolwork and did not have any interests. She finished high school with low grades, partly because she had a depressed period after a break-up with a boyfriend. She dropped out of university after a few months. Later, she moved in with a friend and tried four times to start an education but dropped out repeatedly.

She was assessed in the mental health services under the diagnosis of social anxiety and started psychotherapy in group sessions but withdrew because she was unable to be in sessions with others.

For 3 years she had experienced the presence of a male person sitting on her shoulder, talking into her ear with criticizing statements about her and giving her commands that she felt obliged to obey. Still present, and reaching back as long as she could remember, she had felt the constant presence of yet another person and described herself as spiritually “open to anything.” She never felt that she had any real purpose or meaning in life and often questioned a lot of things seemingly without any special relevance to her: “Why do we have doors, is that really smart? Or who invented the door? Is the color of the door the same to me as it is to you?” Often, she would hear her name being shouted out loud. On a daily basis she experienced thinking about random experiences that seemed to be of no specific relevance to her, but nonetheless would play over and over again, even though she tried to get rid of them. Such thoughts would often play out like an inner picture or movie. She wondered if the world was real or “just a dream in some way,” and when standing on the railway station she might ponder if the people in the train would still exist, when she could no longer see them.

Auditory hallucinations and delusions had thus been present for more than 3 years. Currently, she experienced ongoing vague auditory hallucinations and negative symptoms. Additionally, she described *Anwesenheit*, a diminished sense of basic self, loss of common sense, social anxiety, primary self-reference, ambivalence, perceptual disturbances, mirror-phenomenon, hyperreflectivity, and solipsistic experiences. Her EASE score was 19, PANSS 37, GAF-S 50, and GAF-F 45. Best estimate lifetime diagnosis: Paranoid schizophrenia.

#### Case 2

A 22-year-old male grew up with his mother, who suffered from periodic depression, and he only saw his father on rare occasions. In school, he had anger management issues, but later he became more withdrawn. He had social difficulties and ended up playing computer games and not attending school. He moved to live with his mother’s sister due to problems at home. At age 16–18, he was seen in a child and adolescent psychiatric outpatient clinic and was diagnosed with an attention deficit disorder. Medication helped somewhat on his concentration in school. He went to boarding school, where he had one close friend. He was seen twice in the psychiatric emergency department with suicidal thoughts during this period. He was enrolled in a vocational track in higher education but never showed up. The job-counseling center arranged a traineeship in a shop, which he attended for 4 months but with frequent absence—“then the whole thing went haywire.” He was diagnosed with depression at age 19 and 1 year later he was hospitalized after a suicidal attempt and diagnosed with acute stress reaction.

Social interactions scared him, as he feared to catch people’s attention. He described having many scary thoughts which he initially refused to elaborate on, but whose physical location he showed by placing his hand at the back of his head. After a break in the interview he described further that those “scary thoughts” placed in the back of his head included hearing an ongoing voice, which was not his own, talking to him and addressing his appearance in a devaluating way, always naming him in second person, for instance “you are a bad person.” The voice was described as being “just on repeat.” In addition, he described the voice as being out of his control which scared him. He was not able to reflect further on the experience and in the following just referred to it as “bad thoughts.”

He described auditory hallucinations for at least several months, perhaps longer. He did not describe symptoms of affective disorder, bur harbored pronounced negative symptoms. Additionally, he described spatialization of experience, hyperreflectivity, social anxiety, self-reference, and thought pressure, but had major difficulties verbalizing his psychopathological experiences. His EASE score was 5, PANSS 56, GAF-S 45, and GAF-F 41. Best estimate diagnosis: Non-organic Psychosis Not Otherwise Specified.

## Discussion

We examined a NEET population recruited from outside the mental health services for psychopathology and found that almost all participants qualified for a diagnosis of a mental disorder and that schizophrenia spectrum disorders were the most common. The 53% of the participants, who suffered from a schizophrenia spectrum diagnosis, had higher EASE mean scores and lower global functioning than the rest of the sample.

One third of the participants met the diagnostic criteria for an ICD-10 schizophrenia spectrum disorder but they were not in contact with the mental health services. More than half of the participants who fulfilled the diagnostic criteria of schizophrenia spectrum disorders were in contact with the mental health services but diagnosed with disorders outside this spectrum.

This could reflect that the subgroup meeting diagnostic criteria for schizophrenia spectrum disorders in our sample was less symptomatic than many other schizophrenia spectrum samples described in the literature. This is illustrated, e.g., by a low median PANSS total score of 50.0, which is on par with scores often reported at discharge from psychiatric admissions in schizophrenia spectrum cohorts (47, 48). The mean EASE score of 11.8, is lower than found in studies examining patient samples though one study reported a mean EASE score of 15.5. The most recent systematic review reported a total mean EASE score of 20.7 and 19.7 for schizophrenia and schizotypal disorder, respectively (49).

This low level of symptomatology could perhaps be explained by the illness being in an early phase at the time of the assessment in our study. This explanation is in line with the fact that a decline of social functioning often precedes the development of more obvious psychopathology in these disorders (26, 50). However, it is also well-established that a significant proportion of schizophrenia spectrum patients come across as relatively symptom-poor (51). Eugen Bleuler, who famously coined the concept of schizophrenia, explicitly noted that even manifestly ill patients may at a given time harbor—or at least be able to verbalize—rather few clear-cut symptoms. This, Bleuler noted, poses a significant diagnostic challenge, “because the attending physician cannot testify to the presence of mental disease, or because even if he has testified to it, the director of the mental hospital will occasionally release the patient as well or cured to the dismay of the desperate parents” (52) (p. 296). The existence of such symptom-poor schizophrenia spectrum patients, and the diagnostic conundrums they comprise, is addressed by several other classic psychopathological texts and clinical concepts (53–56). For example, Zilboorg described how such vaguely symptomatic patients, suffering from what he termed ambulatory schizophrenia “seldom reach the psychiatrist’s office for many reasons. First, they are considered by both the laity and the medical profession as merely weak people, “poor personalities,” “psychopathic personalities,” whatever these words may mean” (57) (p. 154).

Indeed, as noted by both Bleuler and Zilboorg, an ordinary clinical encounter with such a patient may reveal nothing particularly remarkable and raise no clear suspicion of a schizophrenia spectrum disorder. On the contrary it often takes an in-depth conversational interview that encourages the patient to reflect and elaborate on his experiences—and sometimes prolonged observation—to reach a valid diagnostic evaluation (39). This, however, is not just a time-consuming process, but also one that presupposes a great deal of clinical experience and sophisticated psychopathological knowledge on the part of the interviewer, all of which may be in short supply in contemporary clinical practice (58).

Importantly, self-disorders also play out in the intersubjective sphere. For example, many persons with self-disorders struggle with the experience of somehow being ontologically different that other people, or “not really human” (59) (p. 94) (i.e., diminished sense of basic self in the EASE). It seems to us that struggling with such experiences of profound dissimilarity might make it quite difficult to engage in social activities with others. Another example could be disturbances of the first-person perspective and hyper-reflection that in some cases lead to excessive forms of self-monitoring which operate alongside the person’s engagement with others thus impeding natural, effortless social interaction (60–62).

The population investigated in this study was not primarily help-seeking in the mental health services but was identified based on their claim of welfare benefits. In the Danish context, social caseworkers are the assessors of qualification for such claims and they are responsible for the evaluation of the young person’s current ability to participate in ordinary education or work. If there are coexisting difficulties, e.g., mental health problems, that hinder this process, the young person is entitled to a coordinated effort aimed at alleviating the problem at hand (30).

However, the complexity of this task seems undervalued. The severe mental health problems found among the NEET population demonstrate the difficulties that social caseworkers with no psychiatric training are faced with. As such they highlight the inherent problems in the physical and organizational separation of the social and mental health services that might, as pointed out in a BMJ editorial, result in insufficient attention being paid to the “double whammy” of NEET status and severe mental disorder (5, 63).

Our findings of a large proportion of schizophrenia spectrum disorders lend support to the guideline proposed by Meehl that a young individual’s gross underachievement seemingly unexplained by relevant factors should—in and of itself—lead the clinician to suspect a schizophrenia spectrum disorder (64). It should prompt a comprehensive differential-diagnostic evaluation as a first glance paucity of overt symptomatology does not rule out the presence of serious mental illness. As noted by Hoch: “A snake in the grass is not a “latent” snake nor a “larval” snake; it is a snake. And it is not concealed by the grass if one looks for it” (65) (p. 724).

This point seems of particular importance to the NEET population, which may be retained in an already disadvantaged position by ineffective psychiatric treatment and inaccurate evaluations in the job-counseling service, if psychotic disorders are not properly detected.

### Strengths and limitations

The principal strength of this study is the comprehensive phenomenological interviews performed by an experienced clinician and the consensus ratings of the psychopathological material. Further, the study was carried out with few exclusion criteria in the real-life setting of a job-counseling center thus enhancing external validity. The ratings were conservative and if it was doubtful if items were present, this item was consistently rated as absent.

The main limitation, on the other hand, is the small sample size. This is a feature of the study’s design with lengthy interviews but also reflects its setting and target group. The study did experience recruitment difficulties with inefficient invitation by the caseworkers among the eligible participants in addition to difficulties in establishing contact with those eligible participant who had accepted the invitation to join. Former studies have described similar difficulties when recruiting among the inactive youth population (66, 67).

It cannot be ruled out, that there was a selection bias built into the invitation procedure of the study, with the caseworkers perhaps being more prone to invite participants whom they believed to be in need of psychiatric assistance. However, the possible effect of such a bias is not univocal. It could be that those not invited were more difficult to reach with an even lower level of functioning and thus, assumingly, an even greater risk of severe mental health problems. Investigating the NEET youth population in a job-counseling center could itself have a built-in selection contributing to the findings of low levels of obvious symptomatology. One could suspect that an even more disadvantaged NEET population with more severe symptomatology might never reach the job-counseling center.

Including NEETs that were already known to have mental disorders in this study did affect the results i.e., a small part of the sample was already previously diagnosed with schizophrenia spectrum disorders. On the one hand, excluding the subjects that were previously diagnosed with mental disorders might have given a more accurate estimate of unrecognized mental disorders. But on the other hand, we found some of the schizophrenia spectrum disorders to be unrecognized among NEETs who had been misdiagnosed with disorders outside the spectrum. Another limitation was the inclusion criterion requiring participants to be fluently Danish speaking. However, this was considered a necessary means to ensuring meaningful participation in in-depth phenomenological interviews.

## Conclusion

The psychiatric examination of a NEET youth sample in a job-counseling service setting revealed that 95% qualified for a diagnosis of any mental disorder. More than half of the sample was diagnosed with a schizophrenia spectrum disorder. The schizophrenia spectrum disorders among the studied sample had a symptom-poor presentation with low PANSS and EASE scores. These findings suggest that pronounced social disconnection in youth—in and of itself—should lead to the suspicion of a serious mental disorder.

## Data availability statement

The raw data supporting the conclusions of this article will be made available by the authors, without undue reservation.

## Ethics statement

The studies involving human participants were reviewed and approved by the Danish National Committee on Health Research Ethics, journal no. 18-000080/Danish Data Protection Agency, journal no. REG-064-2018. The patients/participants provided their written informed consent to participate in this study.

## Author contributions

LL collected the data, performed the statistical analyses, and wrote the first draft of the manuscript. LL, LN, and JN performed the data management. JN and LN contributed to the writing of the manuscript. All authors participated in designing the study, contributed to the revision of the manuscript, which in its present version has been approved by all authors.
